# Capturing Argument in Agent-Based Models

**DOI:** 10.1007/s11245-025-10215-2

**Published:** 2025-06-06

**Authors:** Leon Assaad, Rafael Fuchs, Kirsty Phillips, Klee Schöppl, Ulrike Hahn

**Affiliations:** 1 Munich Center for Mathematical Philosophy, LMU, Geschwister-Scholl-Platz 1, 80539 Munich, Bavaria Germany; 2 Graduate School of Systemic Neuroscience, LMU, Großhadernerstraße 2, 82152 Planegg-Martinsried, Bavaria Germany; 3 Birkbeck College, University of London, Malet Street, London, WC1E 7HX UK; 4 Faculty of Philosophy, University of Groningen, 9712 GL Groningen, Netherlands

**Keywords:** Argumentation, Social epistemology, Agent-based modelling, Bayesian epistemology, Dialectics

## Abstract

Agent-based models (ABMs) are widely used to study the complex dynamics and emergent properties of systems with many interacting agents. This includes belief and opinion dynamics as are of relevance to understanding contexts as varied as online social media and the practice of science. This paper argues that such ABMs can capture rich argumentation scenarios in ways that have not been covered in research to date. To clarify the space of potential agent-based models of argument, we distinguish three interrelated notions of argument from the literature. First, *arguments as reasons* refer simply to the propositional content encoded in arguments. Second, *arguments as syllogism* describe premise-conclusion relationships that arise between such reasons when asserted as arguments. Third, *arguments as dialectics* refer to the deployment of reasons and syllogisms in discussions (be they polylogues or dialogues). We show how modelling each of these three notions of argument naturally involves a continuum of complexity. Specifically, we use the NormAN framework (introduced in Assaad et al. *A Bayesian agent-based framework for argument exchange across networks.* https://doi.org/10.48550/arXiv.2311.09254, 2023), which bases ABMs on the theory of Bayesian networks, as a point of reference and draw out its relationship to other modelling frameworks along each of these dimensions. This provides a novel organising scheme to aid model comparison and model choice, and clarifies ways in which these three notions of argument constrain one another. This shows also that NormAN’s Bayesian framework not only captures familiar facets of argumentation, but also allows one to study how dialectical considerations influence population level diffusion of arguments (as we demonstrate with a small simulation study).

## Introduction: The Importance of Adding Argument to Agent-Based Models

Argument is a tool that mediates much of our human endeavour. Discussion, debate, and deliberation are the engine that drives everything from negotiating our everyday social world, through science, to the political sphere. Unsurprisingly, then, argument and argumentation, are topics of interest across a wide range of theoretical and practical fields. By the same token, the tools used to study argument include qualitative analysis, the evaluation of observational data (in increasingly large quantities such as big data derived from online platforms), behavioural experiments and interventions, and computational modelling (broadly construed).

Computational studies of argument have themselves included everything from formal analysis using the tools of classical and non-classical logics (Prakken and Vreeswijk 2001), the implementation of computational systems that produce or evaluate argument (Gordon et al. 2007; Zukerman et al. 1998; Kowalski and Toni 1997), argument aggregation through standardised representation frameworks (Rahwan and Reed 2009), the implementation of argument exchange in autonomous agents (Rahwan et al. 2009) and, most recently, large language models (e.g., Betz 2022, for an overview see also Hahn and Tešić 2023).

There has also been increasing interest in modelling argument exchange in agent-based models (ABMs in the following). Agent-based modelling is a tool for understanding emergent behaviours in systems of (often large numbers of) interacting agents, whether these be flocks of birds or economies (Helbing 2012; Wilensky and Rand 2015). In particular, agent-based modelling has been used to study group level phenomena such as opinion dynamics (Hegselmann and Krause 2002), polarisation (Olsson 2013; O’Connor and Weatherall 2018; Michelini et al. 2023), or interactions between network structure and belief accuracy in collectives of agents (Zollman 2007, 2010; Hahn et al. 2018a; Mohseni et al. 2021).^1^

The majority of research on belief and opinion dynamics to date has involved agent-based modelling of the propagation of a single numeric quantity (representing opinion strength or a degree of belief) as opposed to the exchange of opinion or belief supporting reasons. It has been known for a while, though, that the dynamics of models that incorporate supporting reasons are substantially different from those of agent models built around the propagation of a single numeric quantity (Mäs and Flache 2013), and there has been increasing interest in building more sophisticated ABMs of argument exchange.

But what exactly should an agent-based model of argument exchange contain? Mäs and Flache’s (2013) model captured ‘arguments’ as plus and minus coded elements in a vector (for similar approaches see e.g., Baccini et al. 2023; Dietrich and Spiekermann 2024). Others model ‘arguments’ as nodes in a graph (Borg et al. 2019; Assaad et al. 2023; Kopecky 2022). Most recently, there has been a surge of agent-based models involving agents able to generate natural language argument (e.g., Betz 2022; Park et al. 2023; Vezhnevets et al. 2023; De Marzo et al. 2024; Chan et al. 2023).

What *is* ‘argument’ and, hence, what kinds of things should a model of argument contain? This is the question pursued in this paper. Our aim with this is twofold: both to further elucidate theoretical notions of argument and to help guide the development of computational systems. To this end, the paper proceeds as follows. First, we review three distinct, but interrelated, notions of argument contained in the literature: arguments as reasons, arguments as syllogisms, and arguments as dialectical exchanges. These three notions provide the conceptual frame for our analysis. We then draw on a recent agent-based model of argument exchange (NormAN, by Assaad et al. 2023) and examine how its features match up to these conceptual distinctions, examining both what is present and what aspects of argumentation are left out.

## The Three Notions of Argument

The literature on argumentation has distinguished three, inter-related meanings of the term ‘argument’ (Hornikx and Hahn 2012).

The first sense of the term ‘argument’ is the notion of argument as a ‘reason’. Providing an argument for something is supplying a reason for it. This notion of argument as reason is applicable whether the something in question is a belief, an opinion, or a recommended course of action. It is exemplified by the reason *the X-ray showed a shadow on the lung* as an argument for accepting the claim *the patient has tuberculosis*. Historically, argumentation research long revolved around logic, and the strongest such reason possible is one from which a claim or conclusion follows by necessity. Many different (non-logical) types of support, however, are found in everyday discourse, and argumentation research has been interested in characterising these types and understanding their impact and prevalence (Pollock 1987; Dutilh Novaes 2022). For ease of reference we will refer to argument in this first sense with the phrase ‘argument as reason’ in the following.

The second sense of the term argument is closely related: the term is now used not just to refer to the reason itself but to a core unit of reason and claim. This is the sense in which ‘logical argument’ is commonly understood: namely as a structured unit comprising one or more premises and a conclusion. These units are widely referred to as ‘syllogisms’, so we will use the phrase ‘argument as syllogism’ in the following to distinguish this from the other two senses. No restriction to logical relationships is intended by this, and an example might be the following two premise argument with associated conclusion:

1. the patient’s X-ray shows a shadow on the lung
2. the patient has recently travelled to a location where tuberculosis is prevalent **therefore**, the patient has tuberculosis

Arguments as syllogisms can refer to minimal structured units and to longer sequences of individual claims, counter-claims, and supporting statements. A well-developed argument on a topic of moderate complexity will typically involve more than a simple, single reason. Argumentation research has developed multiple frameworks such as that of Toulmin (1958/2003) to describe these various relations not just between premise and conclusion but between different premises as well.

Complex syllogistic arguments bring a third, and final, sense of the term argument into view. The term argument refers not just to information content (whether single premise-conclusion pairs or sets of interrelated claims and counter-claims), but also to the social activity, the dialogue or communicative exchange, that gives rise to that content. So, for example, the preceding example of premises and claim, might be information content extracted from a dialectical exchange between multiple parties:

Alan: I’m worried about your cough, you’ve had it for a while.
Beth: Oh, I’m sure it’s nothing.
Alan: You’ve had it for months, you know something like that could be lung cancer.
Beth: It’s not like I ever smoked.
Alan: Well, I think you need to get an x-ray.
Beth: I haven’t got time. And, anyway, I’d be more worried that I have tuberculosis. It’s on the rise where I recently was in Asia. Remember, I told you about that news article last week.
Alan: I just think you should go; there’s enough there to be worried. Better safe than sorry.

For clarity, we will refer to this sense of the word argument as ‘argument as dialectics’. Argumentation researchers have distinguished different kinds of dialectical exchange, ranging from ‘quarrels’ to rational debate (see e.g., Van Eemeren and Grootendorst 2004; McBurney and Parsons 2002a).

Each of these have different characteristics. Rational argument or deliberation, for example, is characterised by both procedural rules (e.g., Van Eemeren et al. 2013; Mischo 2003) and content norms that must be respected for a particular discourse to qualify. Ridiculing an opponent or persuasion via subliminal messages, for example, would plausibly be ruled out. It is this kind of rational discourse broadly that is the focus of our paper, though our analysis does not hinge on precise delineation. Rather, our concern is what aspects of argument can be found in specific ABMs that might be classed as loosely involving argumentation. To this end, we examine both extant and possible features of different systems in light of these three interrelated notions of argument. We next introduce an ABM from the recent literature that will feature as a model system.

## NormAN: A Fruit Fly Argument-Based ABM

Though we will touch on multiple different ABMs for argument exchange, the focal point of our inquiry (its ‘fruit fly’ as it were) will be a recent Bayesian framework for capturing argument exchange named NormAN—short for Normative Argument Exchange Across Networks (Assaad et al. 2023). In complexity, it sits roughly halfway between the already mentioned simple vector-based model of Mäs and Flache (2013) at the one end, and networks of ‘communicating’ large language models (LLMs) that generate, and respond to, natural language text (Chan et al. 2023).

Unlike the Mäs and Flache model in which agents exchange valenced arguments represented only by + 1 and − 1, NormAN agents ‘communicate’ about a ‘world’ that has a semantic interpretation and can involve complex relationships between its various components. Those semantics concern the states of propositional or numeric variables, and the inter-relations between those variables are wholly captured by their probabilistic relationships. Unlike in LLMs, there are no natural language outputs, so the system remains stylised and abstract in this regard.

The analytic value of this intermediate degree of abstraction is determined, ultimately, by the kinds of questions the model can answer, in particular, the kinds of questions that are not answered by either simpler or more complex models along that continuum (on the question of good model more generally, see e.g., Helbing 2012; Martini and Fernández Pinto 2017; Thicke 2020). Though our analysis will surface model features that, arguably, inform analytic value, the extent to which NormAN ultimately is, or is not, a useful or ‘good’ model is not the focus of our investigation. Rather our aim is to use the model to investigate further the notion of ‘argument’ itself as it pertains to computational models of argument, and theoretical treatments of argumentation.

**Fig. 1:**
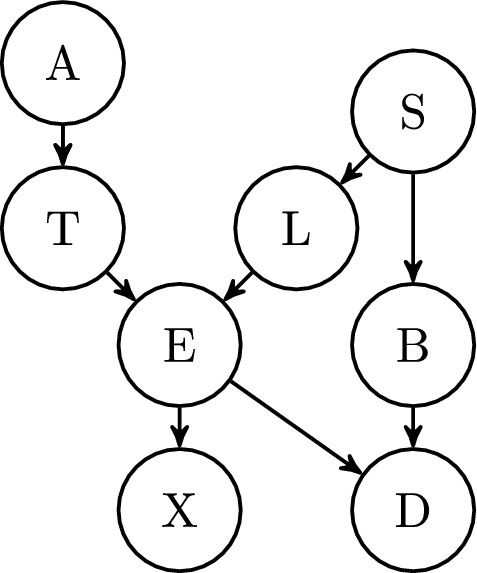
The Asia world. A Bayesian causal graph used to generate an underlying ‘world’ for the simulations in NormAN. Causal variables are whether the patient recently visited Asia (A); has Tuberculosis (T), lung cancer (L), either (E), dyspnoea (D), or Bronchitis (B); shows problematic chest X-ray (X) results; or is a smoker (S). All arrows represent positive probabilistic relevance. (Lauritzen and Spiegelhalter 1988)

To this end, we next describe those basic features of NormAN that are necessary for understanding the subsequent exposition. A simulation in NormAN involves an underlying ‘world’ represented by a Bayesian Belief network (BN) (e.g., Bovens and Hartmann 2003). It supplies the target hypothesis at issue, and the content of potentially available arguments (Fig. 1). Specifically, there is a ‘ground truth’ in any run of a NormAN model, whereby the true state of the target claim at issue is initialised, and a set of possible evidence is created based on that. Agents then receive that evidence either through their own ‘inquiry’ (from the world as it were), or through communication with other agents. At the start of a run, agents are assigned individual pieces of evidence (in the basic version of NormAN, these are chosen randomly, with the number assigned a free parameter in the model). Agents then use their own (veridical) copy of the underlying Bayes’ net to update their belief in the target hypothesis in light of all evidence they have received. The probability of communication at each time step is again a free parameter in the model. When communicating with their neighbours agents choose a piece of evidence from their own evidence list according to a number of possible communication rules, and communicate that piece of evidence faithfully to their neighbour(s). Finally, communication happens across a pre-selected network topology, for example a small-world network (Watts and Strogatz 1998).

The key feature of NormAN, with respect to the notion of ‘argument’, is simply that it revolves around a BN, and ‘arguments’ are (communications of) evidence, that is, variable states in that Bayesian network. Figure 1 shows a sample world BN that is included in the basic version of NormAN (Assaad et al. 2023). It is a causal graph drawn from the literature that represents a network of interrelated variables relevant to the question of whether or not a patient might have lung cancer. The target node is “lung cancer” (L in the figure) meaning the claim at issue on a given model run is whether or not the patient has lung cancer (i.e., the target conclusion/claim at issue is the state of that node as true or false).^2^ The remaining nodes function as evidence. Their values are stochastically generated at the start of a given run (e.g., ‘X-ray’ is set to true, ‘smoking’ is set to false); this set of instantiated variables represents the in principle available evidence in this world. From that set, agents receive items through inquiry or via communication. In other words, they are informed, say, that the patient is not a smoker (if ‘smoking’ is false in the world of that model run), or informed that the patient has recently visited Asia (if ‘asia’ is true on that model run).

One thing to note is that while the BN represents the relationships between variables (e.g., whether smoking supports the presence of lung cancer), it is the actual instantiation (variable state on that run) that ultimately determines whether a given item is evidence for or against the claim. When the value of ‘smoking’ is true, it provides evidence in favour of lung cancer. By contrast, when the value of ‘smoking’ is set to false, it provides evidence against. By that token, the same underlying BN model will give rise to many different instantiated ‘worlds’ and these will vary in the numbers of potential arguments for and against they will contain.

Assaad et al. consider it a virtue of their framework that the model can draw on (somewhat) real-world causal BNs and generate distributions of pro- and con- arguments that are constrained by the underlying structure of the world (Assaad et al. 2023; Hahn et al. 2024). The topic of debate/inquiry and, with it, the individual reasons and their interrelations is, in effect, a parameter in any argument-based ABM, so this is one way in which this important model parameter might be constrained (on the importance of connecting model parameters to the target system, see e.g., Martini and Fernández Pinto 2017). However, as with any type of argument reconstruction, such a BN, even if independently motivated, will still ignore parts of what might plausibly arise in an actual real-world exchange like the snippet between Alan and Beth above. Whether or not Beth is likely to have lung cancer (or better, likely enough to merit concern) is itself only a part of that wider exchange. No model or analysis will include all aspects or parts of such a natural language fragment, simply by virtue of being a model and not a like-for-like duplicate of the actual thing.

Asking to what extent a NormAN simulation captures argument is consequently a question about what aspects of naturally occurring exchanges *could be* included given the level of abstraction and complexity the framework has. We next pursue this for each of the three senses of argument in turn. In so doing, we will also relate NormAN’s position relative to other (computational) approaches to argument.

## In What Ways Does NormAN Involve ‘Argument’?

### Arguments as ‘Reasons’

The first question to address is whether NormAN involves ‘argument’ in the sense of ‘arguments as reasons’. The answer to this, fairly straightforwardly, seems ‘yes’. The individual arguments involved in NormAN are the states of the ‘evidence nodes’. For the Asia network, that corresponds to a proposition such as “The patient does not smoke”. Moreover, these evidence states are actually ‘used’ by NormAN agents to derive degrees of belief in the target hypothesis (“The patient has lung cancer”). This would seem like a quintessential reason relationship (*x* is a reason for believing *y*).

That said, these ‘reasons’ in NormAN are themselves both abstract and restricted, not just vis a vis the full complexity of natural language argument as might feature in discourse analysis, but also in as much as these basic propositional units are un-analysed wholes. There is, for example, no representation of internal logical structure (as captured by the transition from propositional to first-order logic). A propositional representation is clearly a step on a continuum of representational complexity, and an early one at that.

At the same time, the fact that the reasons in NormAN represent propositions suggests that the model does not lend itself to other types of argument content such as opinions (Alan’s “you should go see a doctor”), valuations, body language, or images (Blair 2012). All of these have been of interest to argumentation research. There is, however, a way in which NormAN could be applied by simply changing interpretation. Ultimately a BN is a mathematical object that specifies relations of influence in conjunction with an aggregation rule (Bayes’ rule) that specifies how these influences combine. If one wanted, one could therefore construct BNs that reflect inter-related opinions, for example. What the degree of reason complexity associated with NormAN variables would not allow, however, is to have the same, single reason exert ‘influence’ in multiple ways at the same time (as in an argument that might simultaneously appeal to both head and heart, on the role of emotions, see e.g., Gilbert 2004). Persuasive influence, in NormAN, is restricted to a single channel.

Clearly, the degree of representational complexity NormAN affords in the sense of arguments as reasons is restricted in the sense of excluding at least some real world features of potential research interest and practical relevance. In that sense, what is captured in NormAN is not the fullness of ‘argument as reason’ found in real world contexts. That limitation, in and of itself, does not, however, rule out classifying NormAN’s reasons as argument. There is much research on argumentation that has very productively built on un-analysed reasons that are no more complex than those of NormAN, particularly in computationally oriented work (Dung 1991). So it would seem odd to draw a boundary on what is clearly a continuum of representational possibility at a place that rules out so much extant research.

Furthermore, how limiting a given degree of representational complexity is in practice depends not just on its inherent features but also on the types of relations to other reasons these representations can enter into. We discuss this next.

### Argument as a Unit of Reasons and Claim

Moving on to the notion of argument as syllogism there is likewise a basic sense in which NormAN involves argument. As described earlier, there is a premise-conclusion relationship implied in the fundamental design in that there is a central claim at issue (the true value of the target hypothesis) along with reasons for that claim.

We also see from the basic construction of NormAN around a Bayesian network that the semantic import of arguments as evidence states flows not just from the state of the given variable but from the conditional probabilities that determine its relationship to other variables, both to the target node and to other evidence nodes.

Here, the Bayesian framework used by NormAN is more powerful not just than propositional logic, but more powerful than classical logic as those relationships encompass non-logical relationships as well. Probability, and in particular, the notion of conditional probability, allows one to capture a broad range of relevance relations. These, in fact, subsume logical relationships between different propositions in as much as these can be reflected in appropriate conditional probabilities.

Unlike classical logic, the probability calculus is an intensional calculus: the probabilities assigned follow from the meaning of the proposition, not from logical relationships. It is this feature that gives the probability calculus its value as an analytic tool in the context of Bayesian argumentation (Zenker 2012; Hahn 2020; Dawid et al. 2015). Much of the extant research on Bayesian argumentation was, in fact, driven by the desire to formally capture reason relationships that are present in recurring argument schemes that are found over and over in real world discourse. In the context of fallacies research, for example, the Bayesian framework has been used to help elucidate the strength of particular instantiations (Hahn and Oaksford 2007b). It is used to clarify how different arguments instantiating the same structure or schema (say, argument from ignorance) may be a good argument (say, “the book is in the library, because the catalogue doesn’t say it’s on loan”) or bad (say, “ghosts exist, because nobody has proven that they don’t”).

By the same token, the treatment of (informal) syllogisms in the Bayesian framework (and with that in NormAN) goes beyond not just the qualitative literature on argument, but also much of the computational literature on argument: it provides numerical evaluation. The Bayesian framework allows one to calculate the ultimate, overall, quantitative impact of one or more reasons on the conclusion, that is, how convinced we should be in the claim, given those reasons.^3^ It is thus no surprise that the Bayesian framework has been applied to many contexts involving complex, multi-variate, inferential relationships, whether these be drawn from science (see Howson and Urbach 2006) or law (see e.g., Lagnado et al. 2013; Fenton et al. 2013).

Despite this more general notion of reason relationship, it might seem that there are aspects of ‘arguments as syllogisms’ that are missing: while the relationship between one or more reasons to the *hypothesis* is more general than in many frameworks, the same may not be true of the possible relationships *between reasons*.

In particular, the literature on argumentation has developed multiple frameworks to elucidate relationships not just between claim and reason, but with other reasons as well. For example, Toulmin (1958/2003) distinguished different types of premise material: not just ‘data’ (i.e., facts appealed to for supporting the claim), but also ‘warrants’ (reasons that support the inferential link between data and claim), ‘backing’ (basic assumptions that justify particular warrants) and ‘rebuttals’ (exceptions to the claim or the link between warrant and claim).

Different types of reason relationships also play a central role in more formal approaches to modelling argument. In particular, Dung-style argumentation frameworks are graphs where the nodes represent arguments (without internal structure) and the edges represent attack-relations. Intuitively, if an argument is attacked by another argument, it has to be defended, otherwise it is defeated. Formally, an argument *a* defends another argument *b* against an attacking argument *c* if *a* attacks *c*. There are several definitions for preferences and the acceptability of arguments, building on attack and defense (see Dung 1995). Other frameworks also add edges for explicit support relations between arguments. In particular, the ASPIC+ framework, developed by Modgil and Prakken (2014), aims to make the internal structure of arguments and their different relations more explicit. An argumentation system includes a logical language, a generalised negation function, strict inference rules (where the truth of the premises guarantees the truth of the conclusion) and defeasible inference rules (where the premises support the conclusion, but exceptions are possible). The negation function of the Bayesian framework is more general than the negation used in classical logic, in the following sense: apart from the set-theoretic complement (called “contradictoriness”: *A* and *B* are contradictory, if *A* is in $\overline{B}$ and *B* is in $\overline{A}$, using the overline to denote set-theoretic complement), there also can be “contraries”: *A* is a contrary of *B*, if *B* is in $\overline{A}$, but *A* is not in $\overline{B}$. Finally, (strict and defeasible) inference rules are formulated as schemata with meta-variables (representing arbitrary propositions), where propositional variables must be filled in to instantiate a formula in the language. The classical attack relations of rebuttal and undercut between any two arguments $A_1$ and $A_2$ are then reconstructed as follows:

- Rebuttal: $A_1$ *rebuts* $A_2$ if the conclusion of $A_1$ is a contrary of the conclusion of $A_2$.
- Undercut: $A_1$ *undercuts* $A_2$ if the conclusion of $A_1$ entails the negation of the (*instantiated*) inference rule used in $A_2$.

It seems somewhat under-appreciated to what extent the defeat relations of classical argumentation theory can be directly represented within the Bayesian framework. This has the consequence that different modelling frameworks might seem more different than they actually are with respect to where they lie on the complexity continuum. We thus consider the relationship between argumentation theory and the Bayesian framework in slightly more detail (in addition, the interested reader is referred to the literature on translation from BNs to such argumentation theoretic frameworks, see e.g., Keshmirian et al. 2024).

In order to understand how (generalised versions of) the classical defeat-relations are present in the Bayesian framework, let us look at the classical definitions of rebuttal and undercut:

- A rebuttal attacks by supporting the negation of the conclusion.
- An undercut attacks the inferential link of the original argument (i.e., the original argument is ‘neutralised’).

Here is an example of a rebuttal:

Argument: Tweety is a bird $\Rightarrow$ Tweety can fly.
Rebuttal: Tweety is a penguin $\Rightarrow$ Tweety cannot fly.

Here, additional information leads to the opposite conclusion. This is easily represented in a probabilistic setting: the probability that Tweety can fly if Tweety is a bird is greater than otherwise: $P(F|B) > P(F|\lnot B)$. From this it follows that $P(F|B) > P(F)$, that is, Tweety being a bird supports the conclusion that Tweety can fly. However, if Tweety is a penguin, and penguins cannot fly (by themselves) the probability drops down to zero: $P(F|B,P) \approx 0$.

An example for an undercutter, on the other hand, is the following^4^:

- The object looks red $\Rightarrow$ the object is red.
- The inference (looks red $\rightarrow$ is red) is inapplicable, because the object is illuminated by red light.

Note that the undercut does not lead to the contrary claim, but rather renders the original argument non-diagnostic: that is, the additional information makes the original inference irrelevant. We can model the ‘red object’ example through a simple Bayesian network with three binary (true/false) variables: $R\rightarrow O\leftarrow L$, where *R* represents the hypothesis that the object is in fact red, *O* the observation that the object appears red, and *L* the presence of red light, which also influences the appearance of the object. The argumentative structure outlined above makes use of a particular property of the underlying conditional probability distribution: the fact that a positive instantiation of *L* makes *R* probabilistically irrelevant. We can capture this by the following probability distribution:

- $$P(O|L,R) = P(O|L,\lnot R) = 1$$
- $$P(O|\lnot L,R) = 1 - \epsilon$$, $$0\le \epsilon < 0.5$$ (being generous)
- $$P(O|\lnot L,\lnot R) = 0$$
- $$P(L) = P(R) = 0.5$$

In this case, $P(O) = (2+ 1 - \epsilon )\cdot 0.25 = 0.75 - 0.25\epsilon$ and $P(O|R) = (1-\epsilon + 1)\cdot 0.5 = 1 - 0.5\epsilon$, and hence $P(R|O) > 0.5 = P(R)$, that is, *O* supports *R*.

Therefore, the original argument employs the positive correlation between *R* and *O*, where $O=1$ makes *R* more likely.^5^ However, the undercutter introduces the alternative explanation via red light, which yields $P(R|O,L) = 0.5 = P(R)$, i.e., the original argument is ‘neutralised’ because now $R=1$ is statistically irrelevant to *O*, i.e., the correlation breaks down (supposing the object’s actual colour is overpowered by very strong red light, so the inference doesn’t work). In fact, this is just a special case of what is called ‘*explaining away*’ in the literature on probabilistic reasoning (Tešić et al. 2020).

Importantly, if we generalise the notion of rebuttal, then undercut and rebuttal are continuous: an attack always decreases the probability of the conclusion, and within attacks we can further distinguish undercuts and rebuttals (if we want) as follows: rebuttals lower the probability of the conclusion below its absolute prior (in the extreme case to zero), whereas undercutters are a strong case of explaining away: the undercutter makes the premise probabilistically irrelevant to the conclusion (i.e., the probability of the conclusion given premise and conclusion decreases back to the original prior).

All this is readily captured (and generalised) in the formalism of Bayesian networks.

Finally, the fact that ‘explaining away’ has a natural probabilistic treatment links also to another literature concerned with types of argumentative syllogisms: the literature on so-called argumentation schemes (Walton 2009). Within the literature on informal argument researchers have collected more than 60 different schemes, that is, recurring inferential patterns found in everyday discourse. One aspect of this tradition includes the provision of scheme specific questions, that is, alternative reasons that might undermine the otherwise plausible inference from premise(s) to conclusion on this particular occasion. It has been shown how Bayesian inference and the notion of ‘explaining away’ can capture such inferential relationships (Hahn and Hornikx 2016), providing an alternative to conceptualisations in terms of defeasible modus ponens. Individual schemes drawn from that catalogue have featured in fully implemented computational argument systems (Prakken 2010). They feature also in argument mapping systems like OVA (Lawrence et al. 2019) that support computation across the argument graphs users create. OVA, specifically, offers considerable additional representational complexity. It is possible, for example, to separate out the specific content and the source making the assertion in one’s representation of a reason (Merdes et al. 2021). This, for example, allows one to capture nested chains of assertion as one might find in academic citations (Hahn et al. 2022), where an author might back a claim by asserting, via citation, that another work provides assertion and/or backing of that claim.

Once again, then, we see that NormAN lies on a continuum of syllogistic complexity—a continuum without hard and fast lines or demarcations. But we find also that NormAN, by virtue of drawing on Bayesian Networks, offers greater syllogistic complexity than it might initially seem. This brings us to the third, and final, sense of argument, that of argument as a dialectical exchange.

### Argument as Dialectics

The third, and final, notion of argument highlights that arguments in the first two senses, as individual reasons and as syllogisms, typically arise in dialectical exchanges. This focus on dialectics is a major aspect of argumentation research. It is pursued as a topic of interest in its own right; but it has also been leveraged in attempts to address questions about argument that were long viewed as the domain of argument as reason or as syllogism. For example, broadly dialectic considerations have been harnessed to try to capture aspects of notions such as argument quality or cogency in the context of fallacies research (Walton 1998). Seeking to explain why arguments from ignorance are bad (“ghosts exist because nobody has proved that they don’t”) that cast these as violations of the burden of proof (Walton 2011) invokes a (putative) procedural notion drawn from particular types of dialectic exchange (in this case law, see Hahn and Oaksford 2007a) to support the evaluation of content.

As noted in the introduction, there are multiple frameworks that seek to identify rules for debate and how they might relate to specific argumentative contexts (Van Eemeren and Grootendorst 2004; Mischo 2003). Furthermore, there is the intuitive notion of argumentation as strategically selecting argumentative ‘moves’ that underlies notions of ‘argument as strategic manouvering’ (Van Eemeren and Houtlosser 2006). This has fuelled interest in game theoretic approaches to argumentation (Rahwan and Larson 2009; Matt and Toni 2008; Roth et al. 2007).

Our final question thus concerns the extent to which any of this is (or even could be) reflected in NormAN. To pre-empt our answer, we conclude once again that the dialectical notion of argument, too, involves a continuum. We identify how different aspects of NormAN represent points on that continuum; to do so we examine current features of NormAN as specified by Assaad et al. (2023), alongside subsequent extensions, our own new extensions, and simple (future) extensions. This analysis will show also how the continuum underlying ‘argument’ in the syllogistic sense interacts with what is possible along the dialectical continuum.

Which argument is going to be put forward in a dialectical exchange is going to depend on the argumentative situation, that is, on the epistemic goals of the interlocutors, their backgrounds and (sometimes) also non-epistemic intentions. The literature on argumentation analyses various such situations, each suggesting distinct rules for how arguments should be deployed (Dutilh Novaes 2022): consensus-oriented argumentation, adversarial or cooperative argumentation, argumentation as a truth-conducive epistemic practice and more (see also McBurney and Parsons 2002a).

Contemporary internet debates reveal that, regardless of participant intentions, the nature of communication channels imposes practical constraints on how they can engage. Technical and social constraints give rise to different affordances. These, in turn, give rise to differences in real-world dialectical complexity. For example, presenting an argument on Twitter (X) to an audience of dozens or hundreds of anonymous readers—whose detailed reactions largely remain unknown to the author—differs greatly from the interactive, face-to-face exchanges that classical argumentation theory typically considers the standard. Furthermore, rather than in dyads, argumentation often happens in groups, where group size and communication structure are defined by the given situation (Lewiński and Aakhus 2014).

Let us call combinations of the interlocutors’ intentions (cooperative, epistemic, etc.) and the practical constraints set by the communicative setting, an *argumentative context*. We next show how the NormAN framework can—in principle—capture many of these contexts in an ABM framework. NormAN’s dialectical complexity is thus not fixed. It can be varied. This, in turn, opens up new possibilities for argumentation (and ABM) research: Running simulations can help to inform understanding of what kinds of context will bring forth which types of argumentation dynamics and, ultimately, belief patterns.

#### Selection Rules as Filters

So far we have not described in any detail how NormAN agents go about picking their communications. We have merely noted that, in order to ‘communicate’ with their neighbours, agents choose a piece of evidence from their own, current, evidence list according to a number of possible communication rules. They then communicate that piece of evidence faithfully to their neighbour(s). The dialectical dimension of argument in NormAN thus resides in those *selection rules*.

The most basic rule in the Assaad et al. (2023) implementation is random-share; as the name suggests, it has agents simply randomly selecting an item to communicate from their evidence list. This seems like the simplest selection rule possible. And while it is useful methodologically as a baseline for comparison, it seems so simple as to barely constitute ‘argument’ in dialectical terms.^6^ However, Assaad et al.’s baseline model also has two further selection rules. One is called recency and is sensitive to preceding steps in the discourse (inspired by Mäs and Flache 2013). The other is called impact-share and provides a simple implementation of agents choosing the item that was most impactful in shaping their current belief. We defer any detailed discussion of the nature of these rules in order to draw out an underlying, common, characteristic.

These different communication rules essentially implement different *filters* on the evidence list in order to constrain and prioritise the relative availability of arguments for selection. Such filters can make reference to any number of dialectically relevant criteria: the polarity (valence) of an argument as for or against the hypothesis; its relative strength; its relation to the credence of the speaker; or the status of previous mention in the preceding discourse. Furthermore, any of these criteria (i.e., basic filters) can be combined to create ever more complex filter combinations. Defining increasingly more complex filters makes agents’ communications increasingly more dialectically fine-tuned.

In other words, there is once again a continuum: now with respect to dialectical complexity. Or, more precisely, there is a combinatorial space of basic filters that gives rise to a partial order. We next describe relevant examples in somewhat more detail. First, items can be filtered in terms of polarity. Two possibilities arise here, depending on whether polarity is defined relative to the truth or falsity of the target claim, or whether it is defined relative to the agent’s own current belief:

- Argument polarity: Does a proposition (*E*) constitute an argument in favour of, or against the hypothesis (*H*)? In Bayesian parlance: is its likelihood ratio (LHR) $\frac{p(E|H)}{p(E|\lnot H)}$ larger or smaller than 1? That is, does updating on the argument in- or decrease one’s degree of belief in the hypothesis, if examined in isolation? The latter is a naive version of argument polarity: as outlined above in Sect. 4.2, conditional on other claims being true, the proposition in question may have a very different LHR. Introducing a new feature (as we do below), makes agents sensitive to such changes, enabling them to perceive arguments in light of dyadic conversations.
- Agent polarity: Is the communicating agent’s belief *p*(*H*) higher or lower than their prior? Contrasted to their initial credence in the hypothesis that matched the base-rate encoded in their subjective Bayes’ net, have the arguments they encountered in- or decreased the agent’s hypothesis-credence?

Secondly, in a network such as Fig. 1’s Asia, different nodes vary in diagnostic value, that is, in strength:

- Argument strength: How much is *p*(*H*) impacted by the evidence parcel? How extreme is the likelihood ratio, how strongly does it differ from 1?

Finally, these filters can be made more or less responsive (and hence dynamic) to changes in current beliefs, to the relations and interactions between reasons as discussed above under syllogistic complexity, and to developing knowledge about audience factors. We provide examples of such combinations next, which demonstrate also how readily dialectical complexity can be adapted in order to capture different argumentative contexts.

#### Being Responsive

When choosing an argument to share, what do agents know about their interlocutor? As outlined above, they might not know anything: they might simply post something on Twitter. However, in face-to-face discussions, interlocutors might, at least, try to remember what has been said before. And they can use this to make inferences about what else the proponent knows or believes: even if I am unsure about my partners’ belief about the hypothesis, I might, for example, reasonably infer that they believe it to be true, if I observe them primarily uttering arguments in favour of said hypothesis. Furthermore, I might have a reason to use as rebuttal against my interlocutor’s last argument. Such inferences could be based (and with that implemented) on the basis of everything from simple heuristics to recursive computations (see also McBurney and Parsons 2002b).

Different levels of access to the interlocutor—none, memorizing arguments or direct signals of belief—will constrain how far agents can go in tuning their selections. Let us call them:

1. Egocentric communication: On the first layer, agents’ choices of which arguments to assert depend exclusively on the evidence parcels they have already observed. We want to briefly highlight three communication rules here:
  random-share: Agents select a random known argument to assert to their interlocutor(s).
  impact-share: Agents share the strongest argument with an argument polarity matching their own agent polarity.
  polqua-share: Agents can rank known arguments according to two axes, argument polarity and argument quality. This flexible sharing rule allows them to strategically select any combination of (matching/contradicting/neutral) polarity and (decreasing/increasing/random) quality; and share the first argument in this ranking with very high probability, cascading down the list with decreasing probability.
2. Communication with memory: Knowing what others have already said.
  sample-share: In this agreeable communication rule introduced in Schöppl and Hahn (2024), agents have a memory of the polarity of each neighbour’s last asserted argument. In order to minimise social friction, they assert arguments of matching polarity back to them, either in one-on-one communication, or broadcast-style by matching polarity with the majority of their neighbours (think Twitter followers).
  Dyadic argumentation-memory: Agents, on a per-neighbour basis, keep track of what they have asserted to or heard from each neighbour. Every pair of agents (e.g., *A* and *B*) maintains the same list, which records what has been sent and received during their face-to-face conversations ($\texttt {argumentation-memory}_{A,B}$). argumentation-memory is not a communication rule in itself but rather an information set on which the subsequent rules are based.
3. Dynamic sensitivity to syllogistic relationships: Based on the reasons already known in the dyadic exchange (argumentation-memory), agents reevaluate the strength and polarity of reasons at each time step. This makes them sensitive to changes in the LHRs of propositions by conditioning on reasons already introduced in the conversation. Two new rules use this feature: First, argue-random prompts agents to share a random piece of evidence from their memory that has not yet been introduced in the dyadic interaction. Second, argue-best has agents ask: “What is my best argument given what has been discussed?” At each step of a conversation, agents share their strongest argument that matches their current polarity. If they have no arguments meeting those specifications, they remain silent. To select their ‘best’ argument, agents use their BN representation to condition on the argumentation-memory and identify which of their remaining arguments would most sway belief towards their position, that is, should most effectively influence their interlocutor. Hence, this rule provides agents with more sensitivity and leveraging of the syllogistic relationships between reasons (as outlined in Sect. 4.2).
4. Communication with access to further information: Apart from agent’s own knowledge and what they have observed directly during the communication process, agents’ communication choices may depend on others’ credences, motivations, or sharing-rules. What information agents have about other agents and their beliefs or intentions is again highly dependent on the argumentation context one seeks to model. In some contexts, agents might have to guess or perform highly complex reasoning to assess, for example, other agents’ beliefs (think, for example, the defendants in a legal trial); whereas in others, those beliefs may be readily available (think card-carrying partisans in a political debate).

#### Enacting Argumentative Goals—Layering Complexities

The four layers just described concern the amount of informational and reasoning resources involved in communication. However, this does not settle the question of which motivations lie behind argument assertion, and how complexly the agents reason from these motivations to their choice of communication rule.

Given Assaad et al.’s (2023) aim of rational reconstruction, agents in NormAN, by default, follow (more or less sophisticated) rules aimed at increasing their community’s accuracy by spreading truthful information. Many real-world contexts, however, include other motivations, such as propagating a specific (possibly false) belief, fitting in with a specific group, epistemically outcompeting the rest of the community, doing as little as possible, and so on. These too can be incorporated.

Even just on the first level (egocentric communication), where communication rules refer exclusively to agents’ own stock of evidence parcels, different agent motivations may lead to more or less complex strategies: random-share for instance, is far less complex than polqua-share, because the latter requires agents to rank each known argument according to polarity and quality. Or contrast impact-share with the alternative of ex-ante biased agents that promote a specific conclusion independently of their own beliefs by sharing exclusively arguments in favour of that pre-selected position, such as the industry sponsored strategies introduced in Schöppl (2025). Even egocentric communication varies in dialectic complexity.

Beyond that, one can make layers (2)—(4) as complicated as one likes: k-level rationality, game theoretic considerations, etc. While there is a hierarchy of complexity here, these are all, ultimately, just different filters. No single complexity level corresponds uniquely to ‘argument’; and one of the possibilities the NormAN framework affords is that it allows one to explore the wider ramifications of different combinations and levels.

**Fig. 2:**
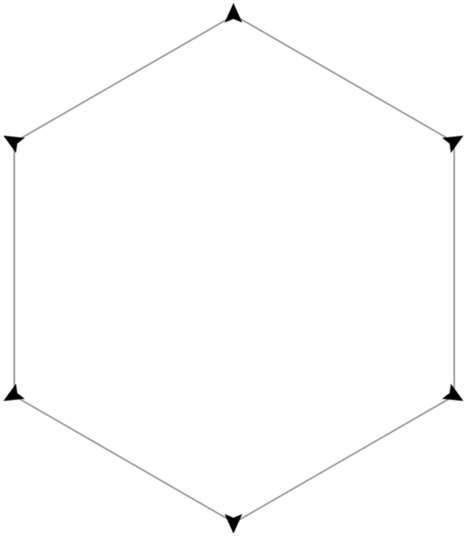
A “cycle” social network of 6 agents as used in Zollman (2007, 2010); Watts and Strogatz (1998)

To demonstrate this, we ran four argumentative scenarios in a simulation, each increasing in dialectical complexity (using an extended version of NormAN).^7^ In each scenario, six agents connected in a “cycle” with two neighbours each (see Fig. 2) debate whether the central hypothesis *L* is true or false. Specifically, they discuss whether a particular patient has lung cancer (*L*), and the BN capturing said deliberation is the discussed Asia network (Lauritzen and Spiegelhalter 1988). Using NormAN, each of the six evidence propositions (i.e., A, T, B, D, S, X in Fig. 1) is randomly initialised as true or false, with each agent initially aware of a different proposition. This exhausts the in-principle available evidence. From this point on, agents merely receive evidence via communication (there is no additional ‘inquiry’); hence, the dynamics are determined solely by communication rules. In the first two scenarios, agents are completely egocentric, and do not respond to each other. The first egocentric group used random-share, randomly sharing evidence, while the second used impact-share, sharing their perceived best evidence. In the later scenarios, agents have dyadic communication with their neighbours, recording what is being said in each neighbour-specific interaction via argumentation-memory. The first responsive group uses argue-random, informing neighbours of randomly chosen arguments that the speaker knows but have not been asserted in the interaction. The second uses argue-impact, selecting the best available argument in favour of their position based on the ongoing conversation (until they had no more such arguments).

**Fig. 3:**
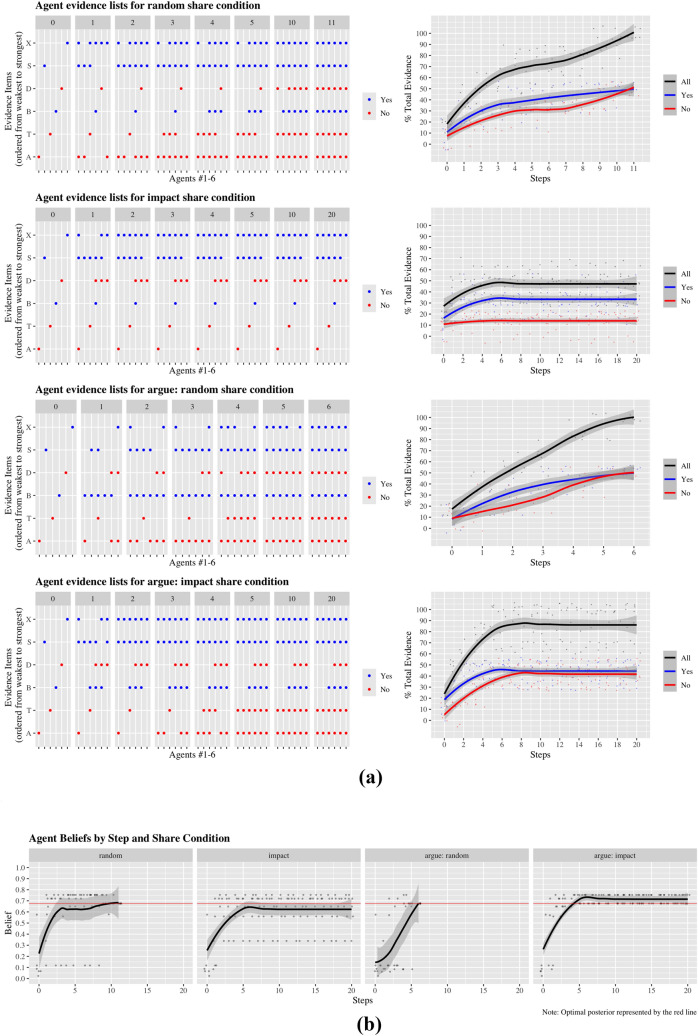
One argumentative scenario, using the Asia network and 6 agents on a cycle network. Each simulation ran for 20 rounds, and the systems reached stability within that time. The simulation code and data are available on OSF: https://osf.io/6kcb9/?view_only=cac360a211df4708b833a81d4f677302

Figure 3 shows how differently these scenarios unfold. Both random conditions lead to full sharing of the evidence, and therefore to a shared belief corresponding to NormAN’s ‘optimal posterior’, i.e., the degree of belief computed based on knowing the entirety of the evidence. However, argue-random achieves this outcome much faster, since a sensitivity to one’s conversational history prevents redundant repetition of arguments. On the other hand, sharing via impact and impact-argue remains incomplete. There, agents only share evidence that serves to convince their neighbours of their current position. As a consequence, some arguments are not disseminated, and agents end up with different, incomplete sets of evidence. Here too, the dynamics created by the more involved impact-argue differ from those arising through the simple impact rule: impact leads to significantly less exchange, resulting in more fragmented sets of evidence and greater differences in final beliefs (cf. Assaad and Hahn 2024 for a study of polarisation via impact share).

The preceding paragraph is not intended to offer an in-depth analysis of the argumentative scenarios, nor is our simulation a comprehensive exploration of the model’s parameter space. This would be beyond the scope of the present paper. However, it does illustrate how the model can be employed to examine the emergent dynamics that follow from combining different argument dialectics (through sharing rules) with different kinds of possible arguments (BNs) in a pragmatic setting (social network): each layering will make for characteristic patterns on the group level, both in terms of argument diffusion and final beliefs. Not only do the different notions of argument interact (i.e., reason complexity constrains syllogistic complexity which, in turn, constrains dialectical complexity), varying these complexities will constrain, via their impact on agent interactions, both the kinds of argument dynamics and belief dynamics that will arise in groups of communicating agents. How such system level dynamics emerge in collectives of argument-exchanging agents is a fundamental question, not just for argumentation research, but also for computational social science, social epistemology, and deliberative democracy. Combining agent-based modelling and complexity science with argument thus opens many new avenues for research.

Finally, we note that there are dialectical moves beyond what we have considered here with our focus on rational argument. These include all interactions that are obviously non-epistemic, such as: aggression, belligerence, or interruption, along with bad faith moves such as deception (as in Douven and Hegselmann 2021), deflection, or twisting of words. These, too, can be analogously layered in terms of setting out the conditions under which they are chosen.

### The Multi-dimensional Space of ‘Argument’

As we have seen, each of the three notions of argument admits gradation, each admits of ‘more or less’. Moreover, the three dimensions interact. The three notions are successively more encompassing as one moves from ‘reason’, through ‘syllogism’, to ‘dialectic’. This translates into constraints on the possible complexity of successive expansions: a very simple notion of ‘reason’ limits the kinds of syllogistic relationships that are expressable, and both, in turn, limit what kinds of dialectical considerations could be captured.

**Fig. 4:**
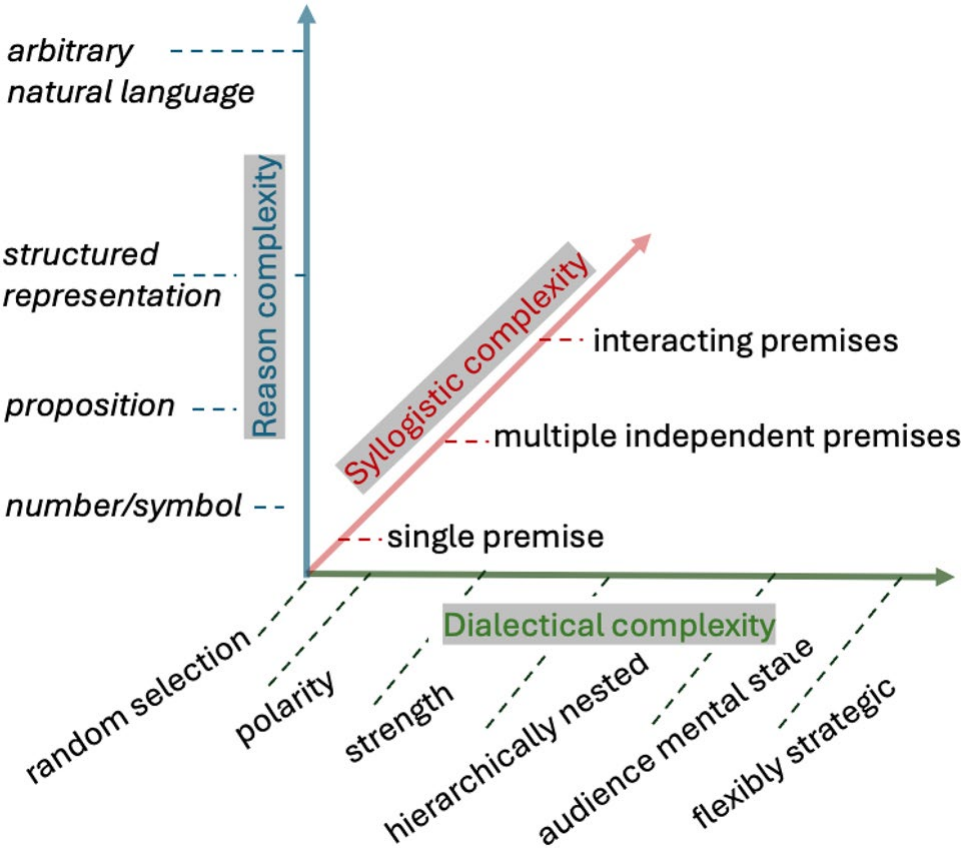
The multi-dimensional space of argument

One can think of the three notions as axes in a multi-dimensional ‘argument space’ where the mutual constraints restrict what points (combinations of values along these dimensions) are, in fact, possible. Figure 4 shows an initial attempt to draw on the various distinctions made thus far and set out such a space. The proviso, here, is that moving along each dimension reflects (at best) an ordinal relationship and the ‘dimension’ of dialectical complexity reflects underlying combinatorial possibilities that themselves are projectable onto a single dimension only with loss.

**Fig. 5:**
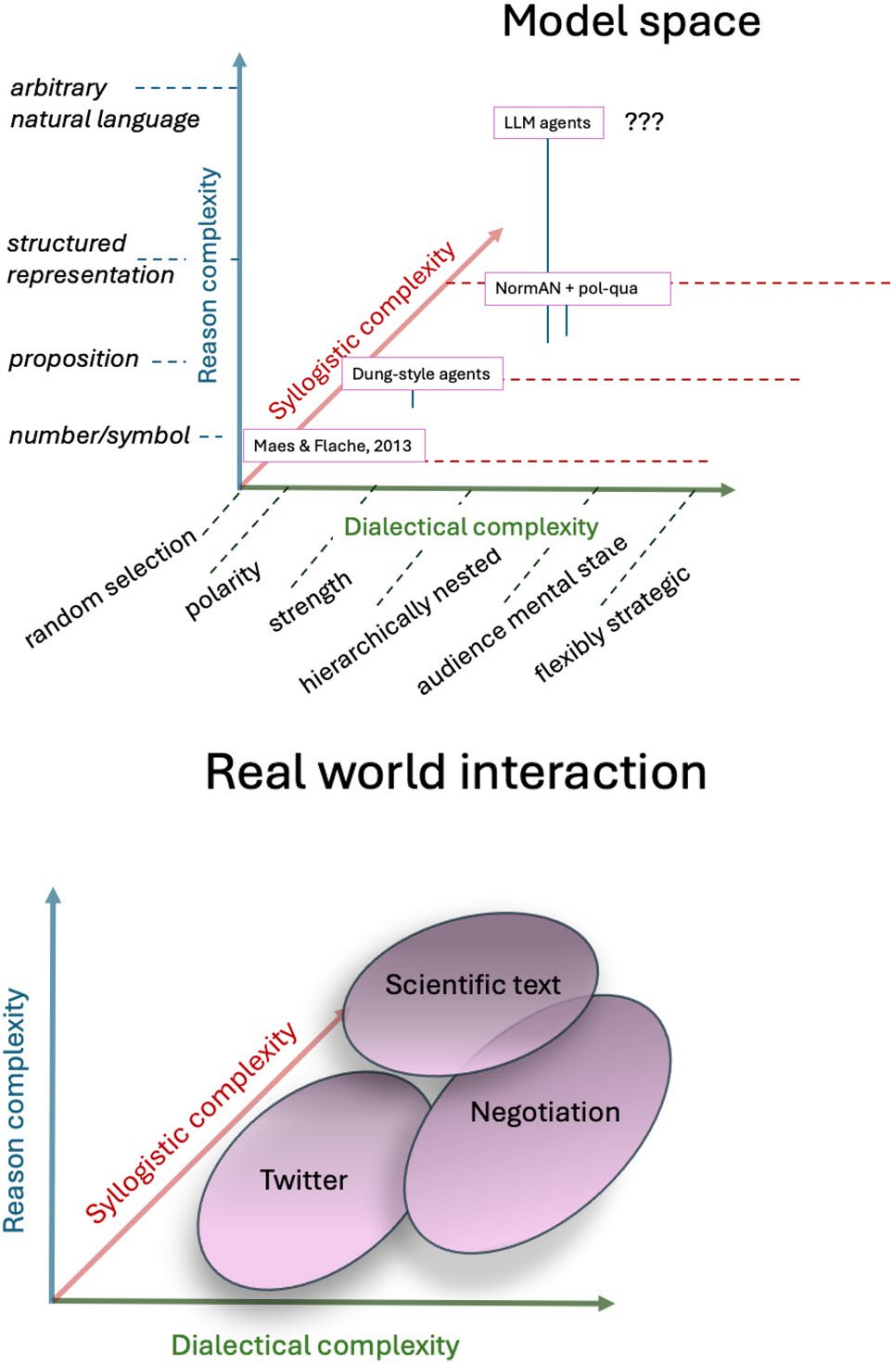
Model space and real-world space. The model space plot situates extant ABMs with argument exchange. The question marks next to LLMs indicate the uncertainty about model capabilities with respect to syllogistic and dialectical complexity. The real world interaction space broadly indicates how different types of discourse vary on the three dimensions of argument complexity. For example, scientific text is arguably higher in reason and syllogistic complexity than most forms of discourse, but, conversely, less sophisticated with respect to dialectical complexity as is, for example, negotiation

Given such a space, we can then also consider how different ABM frameworks relate to one another as in the “Model Space” subplot of Fig. 5.

On a theoretical level, attempting to do this makes clear that the constraint relationship just described does not hold in the opposite direction: complex ‘reasons’ alone do not guarantee syllogistic complexity, nor do either guarantee dialectical complexity. The constraint relationships between the three notions of argument is one of necessity not of sufficiency. This is illustrated by considering LLM-based agents. That they can generate natural language text and thus exhibit maximal reason complexity does not, in itself, guarantee that LLM agents can handle complex syllogistic relationships. Whether they can or not depends on other, additional capabilities of the LLM in question that are to do with its reasoning or inferential abilities [e.g., its ability to handle inductive and deductive arguments (Cheng et al. 2024) its capacity for causal reasoning (Binz and Schulz 2023) or its sensitivity to probabilistic coherence (Betz and Richardson 2023)]. Likewise, dialectical complexity will rest on the extent to which the LLM can select strategies or incorporate recipient mental states (Hagendorff 2024). Alternatively, these abilities may be provided by the LLM embedding agents, in as much as LLM-based ABMs define computational agents that call on a generative language model as a sub-routine (Mitsopoulos et al. 2023). This means also that a superficially ‘simpler’ model in the NormAN family may (along the dimensions of syllogistic and dialectical complexity) show greater argumentative complexity than an LLM-based simulation.

On a practical level, conceptualising ‘argument’ in terms of this argument space may consequently help identify the appropriate degree of complexity for a given analytic purpose. That models should be ‘simple, but not too simple’ is not just an important general principle for modelling, it is particularly pertinent to agent-based modelling. Agent-based models are complex systems, which means emergent behaviours arise often unpredictably from the interactions of individual agents (Ladyman et al. 2013). This can make it difficult to draw robust insights from such models and that endeavour is not helped by extraneous parts.

Finally, one can use such a space to compare different real world contexts of argumentation, see Fig. 5 subplot ‘Real world interaction’. Although all real world argument involves natural language, the language used in different real-world contexts does intuitively vary in complexity (Lu et al. 2019). Likewise, there is variation in the syllogistic complexity of real world arguments seen across different agents and argumentative contexts (Kelly and West 2017). Finally, the dialectical complexity varies (Dascalu 2014). As discussed earlier, it varies as a function of practical affordances (do I know my addressees or not) and functional demand (am I trying to consider different aspects of an issue from a purely epistemic perspective, or am I trying to negotiate a particular action). Additionally, levels of strategising and perspective-taking may also be constrained by individual differences (Conway et al. 2019) and what is deemed a worthwhile degree of effort.

Again, the aim of our paper is not to precisely (or definitively) identify in this space the regions typically occupied by particular argumentative contexts. That is itself a much bigger project. Rather our aim is to draw attention to the way in which different types of real-world argumentative context vary in dimensional complexity. This variability impacts the suitability of particular agent-based models. This variability also informs much more generally the ways in which different research traditions (and methodological toolkits) are more or less suited to different types of argumentative contexts. And, drawing attention to differences in dimensional complexity across different real-world contexts can help identify regions that have been better explored by argumentation research to date and highlight regions that are so far comparatively under-explored. Chief among these under-explored regions are polylogues. One reason for that gap is that polylogues are not naturally handled by treating them merely as an aggregation of dialogues (Lewiński and Aakhus 2014), so dialectical tools that are particularly suited to dialogue are more limited in their methodological value. Agent-based modelling, by contrast, is naturally suited to polylogues and the kinds of differential patterns that emerge as a function of the type of communication rule, as shown in Sect. 4.3.3, are one of the ways in which ABMs bring something new to the study of argumentation.

## What Lies Beyond: Argument Creation, Non-shared Perspectives, (Un)common Worlds

There are additional, further, aspects of argumentation that seemingly lie beyond the just identified ‘argument space’. These aspects, too, are crucial to real-world argumentation, but they might seem orthogonal to the aspects discussed in the paper so far.

### Argument Creation

The first of these is the question of ‘where do arguments come from?’. In NormAN ‘arguments’ are communications about evidence that is ultimately dispensed by ‘the world’. While many of the ‘reasons’ exchanged in fact-oriented debates fit under this general characterisation, it leaves out that agents may have active and strategic roles in the search for that evidence, that is, in establishing those reasons. NormAN models the process of direct (non-communication-based) evidence acquisition as one in which agents simply receive that evidence with no model of what governs that process.^8^ The search for evidence, however, is subject to multiple competing forces (e.g., uncertainty, utilities both in terms of expected pay-offs and resource requirements) that give rise to complex interactions that strongly affect the available evidence base in ways which are often counter-intuitive and difficult to predict. In particular, much research has detailed ways in which seemingly unbiased search strategies can lead to biased collections of evidence (Denrell 2007; Chen et al. 2023) that, in turn, adversely shape the outcomes of collective information exchange and deliberation science. This is an important factor in understanding the kind of belief dynamics that can obtain in groups of communicating agents; it may also be more important to understanding some argument contexts than others. Science likely represents a context where a more detailed model of where the reasons come from is a particularly important factor, so it is likely no accident that much of the work on ABMs modelling communities of scientists have used bandit-models as a simple modelling device for incorporating this aspect (Zollman 2007, 2010; O’Connor and Weatherall 2018; Weatherall and O’Connor 2021).

Relatedly, much psychological work on argumentation can be viewed as examining the antecedent factors influencing people’s ability to produce adequate arguments (including their understanding of what it means to be an argument itself, (Kuhn 1991), and finding means to improve those abilities (Kuhn and Udell 2003; Von Aufschnaiter et al. 2008).

There is also a further sense in which reason content is created in real world argumentation, namely that a reason is itself assembled from more basic evidence (including evidence which is not directly communicated). One example of this in the ABM literature can be found in models of testimony (Olsson 2011; Madsen et al. 2018; Hahn et al. 2019). In these models, the only ‘argument’ communicated is a speaker’s assertion that the claim at issue is true. The nature of that assertion rests on speaker belief: in the Olsson 2011 model, for example, the agent asserts the claim to be true or false when that belief is higher (lower) than a certain threshold (itself a free parameter in the model) and is otherwise silent. This underlying agent belief is itself based on evidence—both evidence ‘from the world’ and other’s testimonial assertions, but that underlying evidence is never passed on by the agent during communication. In this sense, the model involves the communication of ‘reasons’ (an agent asserting “I believe this claim to be true”) that is itself derived, by the communicating agent, from other reasons (private evidence from the world and testimonial assertions received from other agents) that are not themselves things that agent ever communicates.

### Non-shared Perspectives: Source Reliability

This leads on to another aspect of real world argument that lies outside the argument space defined above, namely the connection between argument evaluation and shared perspectives. The Olsson (2011) model just mentioned provides a ready illustration in as much as a major research concern motivating that model and its subsequent use in the literature is the notion of trust. Itself a multi-faceted notion (Pornpitakpan 2004) trust in the Olsson model boils down to the question of the diagnostic value different agents assign to a speaker’s testimonial assertion, that is, how they determine that speaker’s reliability (Merdes et al. 2021). This is a fundamental real world problem in as much as we often have no assured way to estimate the reliability of our sources, and the Olsson model explores a particular Bayesian solution to this problem (Hahn et al. 2018b).

### Non-shared Perspectives: Divergent Worlds

In real-world contexts, different agents may not only have different perspectives on the same reasons, they may also disagree more fundamentally in how they view the world. In particular, there may be divergence in the way they carve up the world, giving rise to discrepant description language. Seemingly the same word may be taken to refer to different things; likewise, one agent’s reasons may involve entities of which another agent hasn’t even heard.

Such divergences are not just possible, they may, in fact, be likely in some of the very arguments we care about most. Feminist epistemology, and standpoint epistemology in particular, has long been concerned with how social location matters crucially for knowledge acquisition (Toole 2019) because it may result in socially situated epistemic privilege, or in socially situated ignorance (Harding 1991; Alcoff 2007). Differences in social identity and the resulting differences in how (well) one understands the world arise any time diverse groups of people come together to exchange arguments, be it in political debate, scientific endeavour or cultural meaning-making through art and media. Incorporating such epistemic diversity in the NormAN framework, which we will tackle in future work, requires outfitting different agents with different, carefully selected subjective causal perspectives (Bayes’ nets) on the world they inhabit; perspectives that reflect their socially situated experiences, knowledge or ignorance.

Case in point, the ‘Asia’ Bayes’ net used above to exemplify the causal relationships between propositions like a patient having lung cancer or tuberculosis, does so by taking a decidedly Western social location as the default perspective—hence why a patient’s ‘recent visit to Asia’ plays the causal role described here. Contrasting this perspective with that relevant to a patient *living in Asia* requires enriching the model with an alternative causal graph that does justice to the respective set of variables and causal dependencies between them.

A simple NormAN model, like those discussed above, will not do justice to these issues by assuming a shared, underlying representation of the world, whether in terms of entities or in terms of diagnostic value.

It will also miss that, even argumentation aside, there exists ample demonstration that human communication often involves the joint negotiation of meaning, such that we jointly form, shape, and agree on the meanings of words within the discourse as it goes along (Clark 1996; Healey et al. 2007). Any simple model of communication as ‘pass the parcel’ will leave out this fundamental aspect of how communication, and with it argument, works.

### Capturing What is Missing

The obvious follow-up question concerning these additional aspects—argument creation, non-shared perspectives, and (un)common worlds—is whether and how these aspects should be included in a particular agent-based model.

The first thing to note is that, as with the dimensions of argument space in their relation to one another, the dimensions of argument space constrain the extent to which these further aspects can be incorporated. What kind of new arguments agents could possibly create depends on the reason complexity and the syllogistic complexity of the model, and dialectical complexity will further constrain how these features can themselves become subject to debate.

For a particular formal framework, such as Bayesianism for NormAN, one needs to consider the extent to which different forms of argument creation, non-shared perspectives and divergent ontologies can be accommodated. This will translate into more technical issues about expanding a model with features like differing subjective Bayesian Networks or ways to expand the algebra from within a Bayesian perspective (Romeijn 2005; Williamson 2003; Romeijn and Williamson 2018; Steele and Stefánsson 2021; Fuchs and Hartmann 2024). It will also require spelling out ways in which agents can argue about BNs themselves (there are already attempt to capture in computational systems the task of co-establishing BNs by agents with competing perspectives that future work might draw on, see e.g., Wieten et al. 2019a, b or arguing despite different BNs Nielsen and Parsons 2007).

On a more conceptual level, however, framing the challenge this way suggests that these missing aspects might themselves largely turn out to be best understood as further facets of the complexity orderings already captured by the basic dimensions. Reasons constructed from other reasons and meta-reasons are, from this perspective, just yet more complex reasons. The same holds for syllogistic complexity: meta-arguments are themselves syllogistic arguments in need of evaluation (on the issue of hierarchically nested dialogue see also McBurney and Parsons 2002b).

Whether this turns out to be the most profitable way to view these issues, it also seems clear that, while they are all important features of (at least some) real-world argumentation, none of these additional concerns seem constitutive of what it means to be ‘an argument’.

Nor is there a sense in which they must be included in any particular treatment of argument. As a fact, they have not been required for most treatments of argument. For example, the strides that have been made in the history of logical analysis and logical approaches to argument have excluded (at least at times) many of these features, and that, arguably, has less been a limitation than a precondition of success. Real-world argument is simply too complex to try and address everything at once; and a model should only ever be as complex as it needs to be. How complex that is flows from the specific goals of a particular modelling endeavour. As noted earlier, all of this is especially true of agent-based models. The value of the conceptual analysis provided here is, ultimately, the extent to which it aids the creation of useful models.

## Conclusions

In this paper, we drew on three distinct notions of the word argument in order to provide a framework for conceptualising the possible ways of implementing argumentation in agent-based models. We distinguished arguments as reasons, arguments as syllogisms, and argument as dialectics. For each, we showed a continuum of complexity with respect to the ways in which that notion might be captured in an agent-based model.

Conducting this examination around a model ABM, NormAN, we showed also how seemingly divergent approaches in the computational literature exhibit deeper correspondence—particularly with respect to the syllogistic notion of an argument.

Furthermore, we illustrated how complex dialectal behaviours (both at individual and collective levels) can be built up by combining simple component filters. We concluded by setting out how the three different notions of argument can be viewed as distinct, but mutually constraining, dimensions of a three-dimensional argument space. Lastly, we showed how this space might be used to position different modelling choices and their implications. This should enable better choices for modellers.

This matters given that there is an urgent need to better understand argument exchange across large scale real world systems such as online social media, in particular. These now occupy an influential epistemic role in human life and evidence of the threats that come with this are accruing (Lewandowsky et al. 2023; Lorenz-Spreen et al. 2023). Agent-based models of argument exchange will be an essential tool for understanding and managing their implications.

## Data Availability

The model code and data sets generated by our simulations are available on OSF, https://osf.io/6kcb9/?view_only=cac360a211df4708b833a81d4f677302.

## Footnotes

1 Confusingly, the term agent-based model of argument is also used to refer to the use of argumentation as a tool for the interaction of autonomous software agents in distributed computing applications, e.g., (McBurney and Parsons 2002a; Castagna et al. 2024). While there are some points of contact to the goals of the current paper, the literature on autonomous agents and our focal literature of agent-based modelling as used in complexity science are largely distinct.

2 The software also allows users to choose other variables as the target node if they so wish.

3 These reasons themselves are contingent and relative, but so is the same thing in logic. We return to this issue in the final part of the paper.

4 An example of an undercut occurring in the familiar Asia net would be the argument that: “The chest X-ray is showing problematic results $\Rightarrow$ the patient likely has lung cancer”, being undercut by the response that “The patient does have Tuberculosis $\Rightarrow$ The X-ray results tell us nothing about the likelihood of lung cancer.”

5 The effect is stronger if *P*(*L*) is low in the first place, but in order to simplify calculations we chose 0.5; the qualitative effect remains the same.

6 Though, of course, arguments’ appearance in the evidence-list already pre-filters for relevant assertions in the sense of argument as reasons, and argument as syllogisms, because propositions only appear in the world Bayes’ net if they are causally relevant to the hypothesis directly, or are relevant when mediated by other propositions. For an application of the NormAN framework that de-idealizes this feature to model the ‘Flooding the Zone’ strategy of political misinformation, see Hahn et al. (in press).

7 Our simulation code and data can be found via this anonymised OSF link: https://osf.io/6kcb9/?view_only=cac360a211df4708b833a81d4f677302.

8 The actual process is summarised by a single parameter governing the probability of ‘inquiry’, which sets the probability that a piece of evidence drawn from the in-principle available evidence is received at time step *t*.
